# Health-related Quality of Life in European Older Adults with Respiratory Syncytial Virus Over Three Respiratory Syncytial Virus Seasons

**DOI:** 10.1093/cid/ciag147

**Published:** 2026-03-06

**Authors:** Manel Terns Riera, Rosa Prato, Alberto Pérez-Rubio, José María Echave-Sustaeta, Shubhangi Gawade, Philip Joosten, Eliazar Sabater Cabrera, Desmond Curran, Eliana Biundo, Daniel Molnar, Frederik Verelst, the RSV-029 study group, Julio Ancochea Bermúdez, Laurence Bocqiua, Georg-Eike Böhme, Carlos Brotons Cuixart, Charles Bundy, Silvia Damaso, Nicolas Depaye, Cristina Genovese, Veronica Hulstrøm, Bastian Kirsch, Damien McNally, Helena Moza Moríñigo, Silvia Narejos Pérez, Carlo Pomari, Saul Robles, Fernando Sánchez Perales, Revathi Thimmaiah, Paul Torres Gutiérrez, Giacomo Tuana Franguel, Els Van de Paar, Claus Von Hessert

**Affiliations:** CAP El Remei, Vic, Spain; Hygiene Unit, Policlinico Foggia Hospital, University of Foggia, Foggia, Italy; Complejo Asistencial de Ávila, Ávila, Spain; Hospital Universitario Quirónsalud Madrid, Universidad Europea de Madrid, Madrid, Spain; GSK, Bangalore, India; GSK, Wavre, Belgium; GSK, Wavre, Belgium; GSK, Wavre, Belgium; GSK, Wavre, Belgium; GSK, Wavre, Belgium; GSK, Wavre, Belgium

**Keywords:** respiratory syncytial virus, health-related quality of life, FLU-PRO, EQ-5D-3L, health utility

## Abstract

**Background:**

Respiratory syncytial virus (RSV) is an important cause of respiratory illness, affecting individuals of all ages. RSV can cause severe disease in older adults, but the disease burden and how it affects the health-related quality of life (HRQoL) is not well characterized.

**Methods:**

This multicenter, cross-sectional study conducted over three RSV seasons (October 2021–April 2024) in six European countries enrolled adults ≥60 years of age (YOA) who presented to general practitioners or outpatient clinics with an acute respiratory infection (ARI) and who were not vaccinated against RSV. The symptom duration, the effect of medically-attended confirmed RSV-ARI (cRSV-ARI) on symptomology (InFLUenza Patient-Reported Outcome [FLU-PRO] questionnaire) and HRQoL (EuroQoL-5 Dimension [EQ-5D-3L] questionnaire) as well as the healthcare resource utilization and working days lost were assessed.

**Results:**

We analyzed 136 participants with a medically-attended cRSV-ARI. The median duration of any ARI symptom was 2.0–19.0 days. cRSV-ARI symptoms mostly affected the chest/respiratory domain (mean score: 1.72; standard deviation [SD]: 0.76) and the nose domain (mean score: 1.58; SD: 1.00). The mean country-specific cRSV-ARI utility scores ranged from 0.81 on Day 1 to 0.90 on Day 29. On Day 57, the score was 0.84. A trend for an increased effect on HRQoL was observed in participants with lower respiratory tract disease. The mean duration of treatment was 16.3 (SD: 11.9) days, and 27.8% of participants in active employment stayed home from work.

**Conclusions:**

medically-attended cRSV-ARIs substantially affect the symptomology and HRQoL of European adults ≥60 YOA who were not vaccinated against RSV.

## Teaser

medically-attended confirmed respiratory syncytial virus (RSV)-associated acute respiratory infections affect the symptomology and the health-related quality of life of European adults aged 60 years and older who were not vaccinated against RSV.

**(See the Editorial Commentary by Branche on pages e1289–91.)**

Respiratory syncytial virus (RSV) is an important cause of respiratory illness [1]. In temperate climates, the two major antigenic groups (A and B) circulate during winter months, while in tropical climates their circulation is more variable [1, 2]. Although RSV affects individuals of all ages, infants, older adults, and people with chronic medical conditions (eg, cardiopulmonary diseases, diabetes, or immunocompromised status) are at increased risk for severe RSV disease [1, 3].

The RSV prevalence and burden of disease have long been underappreciated in adults due to a lack of recognition, ascertainment, testing, and detection [4, 5]. Yet, RSV can cause severe infection or death in adults ≥50 years of age (YOA), especially in those who are frail, have underlying medical conditions, or live in a nursing home [5, 6]. A systematic literature review estimated that the number of RSV-associated acute respiratory infection (ARI) cases in adults ≥60 YOA in high-income countries could reach 5.7 million by 2025, with 510 000 associated hospitalizations, and 37 000 in-hospital deaths [7]. In addition, the RSV disease burden and its effect on the individual's health-related quality of life (HRQoL) and healthcare resource utilization (HCRU) is not well characterized in adults ≥60 YOA [8, 9]. In a cross-sectional study among 30 individuals ≥50 YOA who experienced a polymerase chain reaction (PCR)-confirmed RSV episode, gastrointestinal symptoms were reported by 19 participants, and coughing, trouble breathing, fever or feverish, and body aches or pains were reported as most inconvenient [10].

As of May 2025, three vaccines are available and licensed for use in older adults in Europe and the United States: an adjuvanted protein-based vaccine (adjuvanted RSVPreF3, *Arexvy*, GSK), a protein-based vaccine (RSVpreF, *Abrysvo*, Pfizer Inc.), and an mRNA-based vaccine (mRNA-1345, *mResvia*, Moderna, Inc.) [11–14].

The aim of the present study was to estimate the prevalence and characteristics of RSV-ARI (as disclosed in Terns Riera et al [15]) and to assess the effect of medically-attended confirmed RSV-ARI (cRSV-ARI) on symptomology, HRQoL, and HCRU in European adults ≥60 YOA who were not vaccinated against RSV.

**Figure 1.**
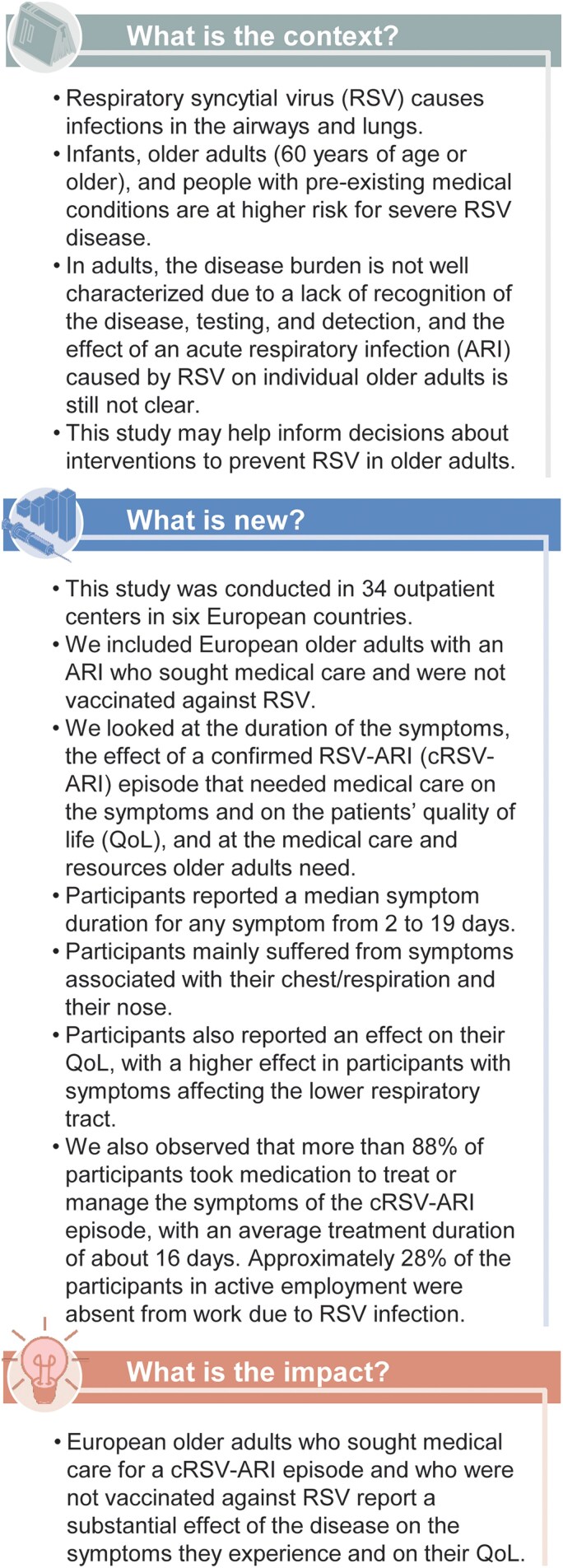
Plain language summary.

A summary of the study findings in plain language is presented in Figure 1.

## METHODS

### Study Design

**Figure 2.**
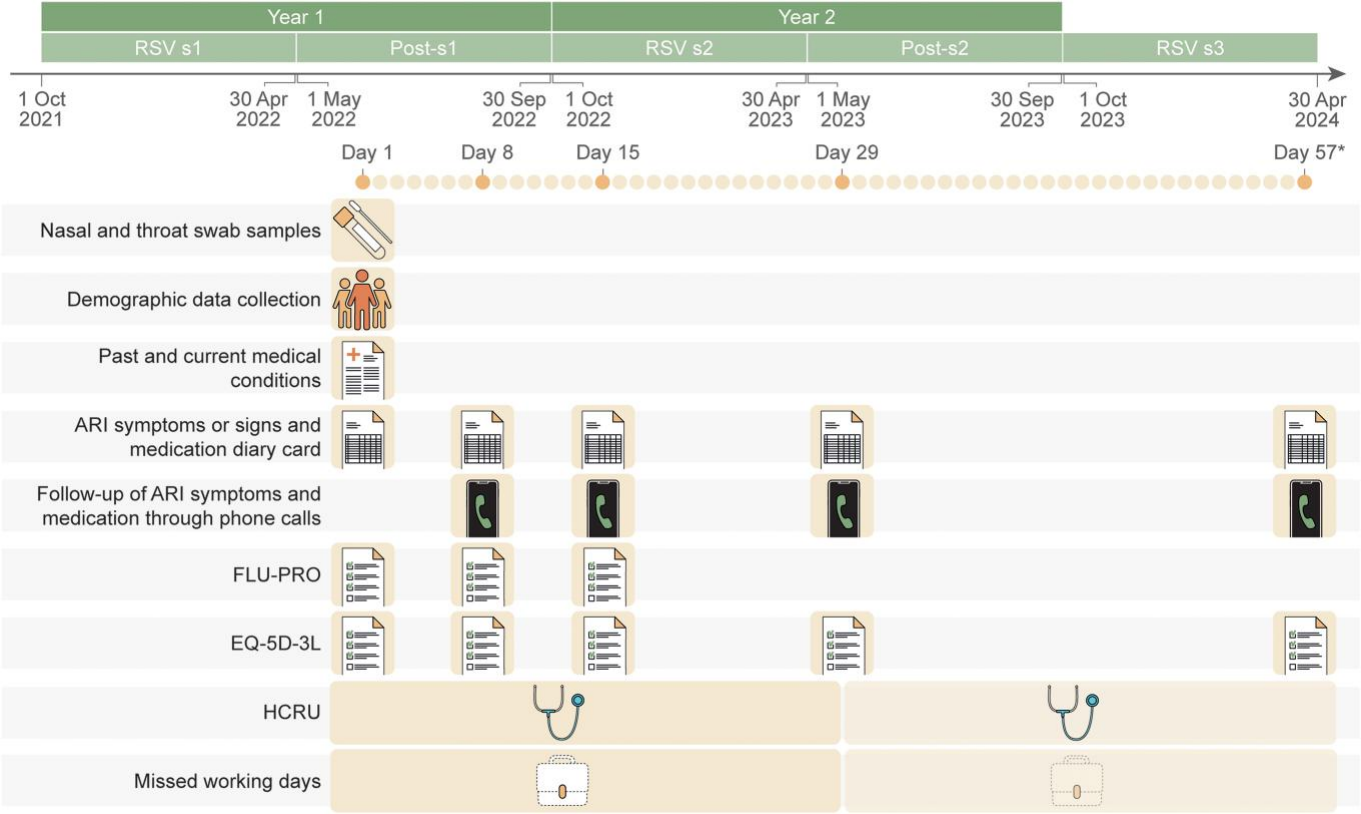
Study design. Abbreviations: ARI, acute respiratory infection; COVID-19, coronavirus disease 2019; eCRF, electronic case report form; EQ-5D-3L, EuroQoL-5 Dimension; FLU-PRO, InFLUenza Patient-Reported Outcome; HCRU, healthcare resource utilization; RSV, respiratory syncytial virus; s, season; RT-PCR, reverse transcription polymerase chain reaction; SARS-CoV-2, severe acute respiratory syndrome coronavirus 2. *Only for participants with symptoms persisting after Day 29. Participants from Finland were only recruited in RSV s1. Participants from Poland and Germany were recruited in Year 2 and RSV s3. Nasal and throat swab samples were collected at enrollment (Day 1) to determine the causative respiratory virus. One swab was used to sample both nostrils of each participant. The second swab was used to sample the throat. The swabs were placed in the same collection tube and sent for analysis using multiplex RT-PCR assays. Demographic data including year of birth, sex, country, race, and employment status were recorded in an eCRF on Day 1. A medical interview to assess past and current medical conditions was conducted on Day 1. Comorbidities of interest were cardiac disorders (eg, congestive heart failure, myocardial infarction, and ischemic heart disease), chronic pulmonary conditions (eg, chronic obstructive pulmonary disease, asthma, and emphysema), diabetes types 1 and 2, cancer, SARS-CoV-2 infection, and the immunocompromised status (due to chronic condition or medication). Vaccination history (influenza, pneumococcal, pertussis, and COVID-19 vaccination) was also recorded in the eCRF. Transcription of the completed questionnaires in the eCRF was performed by the site staff as soon as the participant or their caregiver returned the questionnaire.

This cross-sectional study was conducted in 34 outpatient centers in six European countries (Finland, Germany, Italy, Poland, Spain, and the United Kingdom) between October 2021 and April 2024. The study thus covered three consecutive RSV seasons. Respiratory viruses were detected by reverse transcription polymerase chain reaction (RT-PCR), as described by Terns Riera et al [15]. An overview of the study design is presented in Figure 2.

The study was conducted according to the protocol, ethical principles derived from international guidelines including the Declaration of Helsinki, the Council for International Organizations of Medical Sciences International Ethical Guidelines, and all other applicable laws and regulations. The protocol and its amendments were approved by the relevant independent ethics committees or institutional review boards, as disclosed in Terns Riera et al [15]. All participants provided written informed consent before enrollment.

### Study Population

The study included adults ≥60 YOA with an ARI onset date <7 days before general practitioner (GP) or outpatient clinic visit. This period was expanded to 10 days in a second protocol amendment on 20 June 2022 as adults ≥65 YOA shed virus at higher titers and for a longer duration. An ARI episode was defined as ≥2 respiratory symptoms or signs for ≥24 hours or ≥1 respiratory symptom or sign and one systemic symptom or sign for ≥24 hours. A description of the symptoms or signs and detailed eligibility criteria are disclosed in Terns Riera et al [15]. Participants enrolled more than once in the study were considered as independent participants in the analysis sets.

### Objectives

The primary objective (prevalence of medically-attended cRSV-ARI) and most secondary objectives are disclosed in Terns Riera et al [15]. The present manuscript includes the following secondary objectives (1) description of the duration and occurrence of symptoms or signs of medically-attended ARI in participants with cRSV, (2) assessment of the effect of medically-attended cRSV-ARI on patients' HRQoL, and (3) assessment of the HCRU and working days lost by participants with medically-attended cRSV-ARI and their caregivers.

### Outcome Measures

Participant-reported outcomes were collected using two paper questionnaires: the InFLUenza Patient-Reported Outcome (FLU-PRO) version 2.0 and the EuroQoL-5 Dimension (EQ-5D-3L) version 1.0 questionnaires. FLU-PRO questionnaires were completed on Days 1, 8, and 15. EQ-5D-3L questionnaires were completed on Days 1, 8, 15 and 29 if the ARI resolved within four weeks and on Days 1, 8, 15, 29, and 57 if the ARI was not resolved by Day 29 (Figure 2). Paper questionnaires could be returned in person or by mail. If one or more timepoints were missing, the other available timepoints were considered for the analysis.

The FLU-PRO questionnaire is a 32-item daily diary [16] focusing on influenza symptoms and signs across six domains, ie, nose (four items), throat (three items), eyes (three items), chest/respiratory (seven items), gastrointestinal (four items), and body/systemic (11 items) (Table 1). Participants were asked to rate each symptom or sign severity on a 5-point ordinal scale; from 0 (not at all) to 4 (very much) for 27 items; 0 times to ≥4 times for vomiting and diarrhea; and from 0 (never) to 4 (always) for sneezing, coughing, and coughed up mucus or phlegm. Individual domain scores were computed as the mean of all symptoms or signs in the domain, the total score was computed as the mean score across all 32 symptoms or signs, and the maximum score was the highest score observed among the scores from Days 1, 8, and 15. The scores ranged from 0 (no symptoms) to 4 (very severe symptoms). For both the domain scores and the total score, the score was set to “missing” if more than 50% of the items were missing. The validity of this questionnaire to assess RSV in older adults has been previously demonstrated [17].

**Table 1.** FLU-PRO Domain Components

Domain	Symptoms or Signs
Nose	Runny or dripping nose
	Congested or stuffy nose
	Sinus pressure
	Sneezing
Throat	Scratchy or itchy throat
	Sore or painful throat
	Difficulty swallowing
Eyes	Teary or watery eyes
	Sore or painful eyes
	Eyes sensitive to light
Chest/respiratory	Trouble breathing
	Chest congestion
	Chest tightness
	Dry or hacking cough
	Wet or loose cough
	Coughing
	Coughed up mucus or phlegm
Gastrointestinal	Felt nauseous (feeling like you wanted to throw-up)
	Stomach ache
	Vomit (frequency)
	Diarrhea (frequency)
Body/systemic	Felt dizzy
	Head congestion
	Headache
	Lack of appetite
	Sleeping more than usual
	Body aches or pains
	Weak or tired
	Chills or shivering
	Felt cold
	Felt hot
	Sweating

Abbreviation: FLU-PRO, InFLUenza Patient-Reported Outcome.

The EQ-5D-3L questionnaire [18] focusses on health utility in terms of mobility, self-care, usual activities, pain/discomfort, and anxiety/depression. Participants were asked to grade each item from 1 (no problem/no symptom) to 3 (highest level of difficulty or symptom), resulting in 243 different profile combinations which were converted to a continuous single index utility score ranging from 1.00 to −0.111. EQ-5D-3L utility scores could only be derived if all items were answered. Country-specific EQ-5D value sets were used to generate the utility scores. A systematic literature review showed that the EQ-5D-3L questionnaire is applicable in adults ≥65 YOA [19].

HCRU was estimated by collecting information on physician visits, outcome of referral to other medical facilities, emergency room (ER) visits, intensive care unit (ICU) admissions, hospitalizations, treatment, and working days lost by participants presenting with ARI and their caregivers.

### Statistical Methods

Information on the study sample size and the analysis set is disclosed in Terns Riera et al [15]. The analysis set for medically-attended cRSV-ARI cases included all participants from the analysis set with a positive RSV A and/or B RT-PCR test result.

Adherence to the schedule of completion of the FLU-PRO and EQ-5D-3L questionnaires was calculated as the number of completed questionnaires compared to the number of questionnaires that were expected to be completed at each timepoint.

All statistics were descriptive. Two-sided 95% confidence intervals (CIs) were calculated using the extended Clopper-Pearson exact CI for clustered data [20]. For continuous variables, the mean, standard deviation (SD), median (range), and interquartile range was reported. For discrete variables, the number of events and the number of participants were reported. EQ-5D-3L utility scores were also analyzed by lower respiratory tract disease (LRTD) status.

## RESULTS

### Study Participants

A total of 2573 distinct participants were enrolled, of whom 139 had a medically-attended cRSV-ARI. Three participants were excluded from the analysis set due to ineligibility (two participants in Year 1 and one participant in Year 2), resulting in 136 participants. Further details on participant disposition and changes following database lock are disclosed in Terns Riera et al [15].

For the analysis set, the mean age at ARI onset was 68.7 years (SD: 6.9) in Year 1, 69.9 years (7.3) in Year 2, and 70.0 years (7.2) in RSV season 3. Across groups, 57.7%–65.9% of participants were women, and 97.1%–100% were White (European heritage). Demographic characteristics are detailed in Terns Riera et al [15].

### Occurrence and Duration of ARI Symptoms or Signs in Participants with Medically-attended cRSV

The occurrence of ARI symptoms or signs is disclosed in Terns Riera et al [15]. Briefly, 91.9% (95% CI: 83.4%–96.9%) of participants reported upper respiratory symptoms or signs (with 83.1% reporting nasal congestion or rhinorrhea), 99.3% (96.0%–100%) reported lower respiratory symptoms or signs (with 97.8% reporting cough), and 87.5% (76.7%–94.5%) reported systemic symptoms or signs (with 64.0% reporting fatigue).

The median duration of any ARI symptom or sign was 2.0–19.0 days for individual respiratory symptoms or signs and 6.0–17.0 days for individual systemic symptoms (Table 2).

**Table 2.** Duration (in Days) of Each Symptom or Sign Reported During the Medically-Attended cRSV-ARI (Analysis Set)

Symptoms or Signs	*N* with Data^a^	Medically-Attended cRSV-ARI (*N* = 136)
Upper respiratory symptoms	…	…
Nasal congestion or rhinorrhea	109	…
Mean (SD)	…	19.2 (11.5)
Median (minimum-maximum)	…	16.0 (3–57)
Sore throat	87	…
Mean (SD)	…	14.1 (10.6)
Median (minimum-maximum)	…	10.0 (3–48)
Lower respiratory symptoms or signs	…	…
Cough (new or worsening)	127	…
Mean (SD)	…	20.9 (12.6)
Median (minimum-maximum)	…	19.0 (3–58)
Sputum production (new or worsening)	93	…
Mean (SD)	…	21.4 (10.5)
Median (minimum-maximum)	…	19.0 (5–52)
Dyspnea or shortness of breath	…	…
(new or worsening)	76	…
Mean (SD)	…	16.0 (11.4)
Median (minimum-maximum)	…	14.0 (1–58)
Wheezing (new or worsening)	12	…
Mean (SD)	…	4.3 (4.5)
Median (minimum-maximum)	…	2.0 (1–14)
Systemic symptoms or signs	…	…
Myalgia	63	…
Mean (SD)	…	14.4 (9.6)
Median (minimum-maximum)	…	12.0 (1–51)
Arthralgia	44	…
Mean (SD)	…	15.4 (11.0)
Median (minimum-maximum)	…	13.5 (1–56)
Fatigue	82	…
Mean (SD)	…	20.1 (13.6)
Median (minimum-maximum)	…	17.0 (1–62)
Headache	69	…
Mean (SD)	…	15.6 (14.0)
Median (minimum-maximum)	…	11.0 (1–62)
Decreased appetite	47	…
Mean (SD)	…	13.7 (9.8)
Median (minimum-maximum)	…	10.0 (3–39)
Feverishness	43	…
Mean (SD)	…	10.8 (8.9)
Median (minimum-maximum)	…	9.0 (1–38)
Fever (confirmed by temperature measurement of ≥38.0°C or ≥100.4°F)	25	…
Mean (SD)	…	8.8 (9.1)
Median (minimum-maximum)	…	6.0 (1–36)

The occurrence of ARI symptoms and signs is disclosed in Terns Riera et al [15].

Abbreviations: ARI, acute respiratory infection; cRSV-ARI, confirmed respiratory syncytial virus-associated acute respiratory infection; N, number of participants; SD, standard deviation.

^a^Participants with elimination code 2500 (diary card not completed) were excluded from the analysis.

### Effect of Medically-attended cRSV-ARI on Symptomology and HRQoL

In participants with a medically-attended cRSV-ARI, compliance with completion of the paper questionnaires was ≥95.6% at all timepoints.

**Figure 3.**
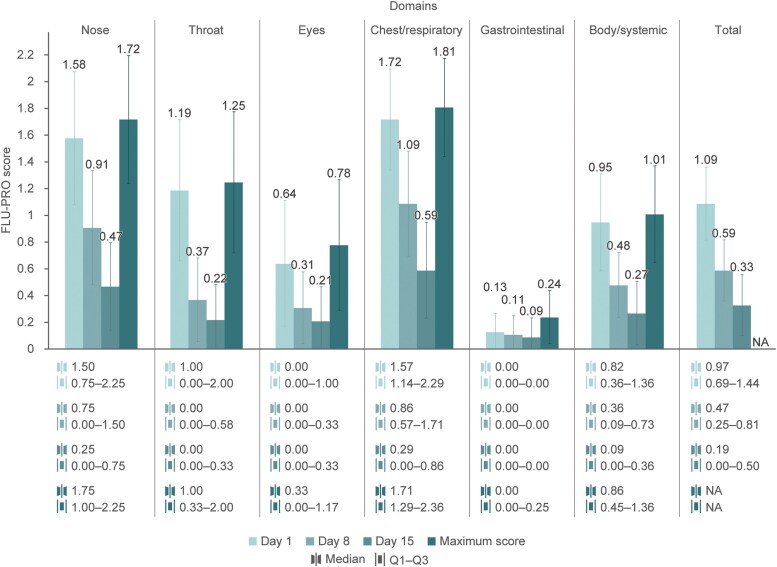
Mean FLU-PRO scores at Days 1, 8, and 15 and maximum score per domain for participants with a medically-attended cRSV-ARI (analysis set for medically-attended cRSV-ARI cases). Abbreviations: cRSV-ARI, confirmed respiratory syncytial virus-associated acute respiratory infection; FLU-PRO, InFLUenza Patient-Reported Outcome; NA, not available; Q1, 25% percentile; Q3, 75% percentile. Error bars depict the standard deviation. Only the portion of the error bars above zero is shown.

Based on the FLU-PRO scores on Day 1, the most affected domains were chest/respiratory with a mean score of 1.72 (SD: 0.76) and nose with a mean score of 1.58 (1.00). The least affected domains were gastrointestinal with a mean score of 0.13 (0.28) and eyes with a mean score of 0.64 (0.95). The mean scores on Day 15 were 0.59 (0.72) for chest/respiratory and 0.47 (0.66) for nose (Figure 3). The maximum FLU-PRO scores in participants with a medically-attended cRSV-ARI were reported for the chest/respiratory (1.81; SD: 0.74) and the nose (1.72; 0.96) domains (Figure 3). Over the different age categories, the mean FLU-PRO scores for the chest/respiratory domain ranged from 1.53 to 1.82 on Day 1, from 0.95 to 1.25 on Day 8, and from 0.39 to 0.79 on Day 15 (Table 3). For the nose domain, the ranges were 1.48 to 1.80 on Day 1, 0.56 to 1.23 on Day 8, and 0.31 to 0.64 on Day 15 (Table 3).

**Table 3.** FLU-PRO Scores at Days 1, 8, and 15 and Maximum Score per Domain for Participants With a Medically-Attended cRSV-ARI by Age Category (Analysis Set for Medically-Attended cRSV-ARI Cases)

			Domains						
			Nose	Throat	Eyes	Chest/respiratory	Gastrointestinal	Body/systemic	Total
60–64 YOA	Day 1	N with data	37	36	37	37	37	37	37
		Mean (SD)	1.52 (1.00)	1.31 (1.06)	0.67 (0.91)	1.69 (0.81)	0.18 (0.34)	1.12 (0.81)	1.15 (0.59)
		Median	1.25	1.33	0.33	1.43	0.00	0.91	1.06
		Q1–Q3	0.75–2.25	0.67–2.00	0.00–1.00	1.14–2.14	0.00–0.25	0.55–1.70	0.81–1.44
		Min–Max	0.0–3.8	0.0–4.0	0.0–3.0	0.4–3.7	0.0–1.3	0.0–3.2	0.2–2.9
	Day 8	N with data	35	35	35	35	35	35	35
		Mean (SD)	1.06 (0.87)	0.42 (0.68)	0.29 (0.36)	1.13 (0.82)	0.12 (0.30)	0.53 (0.51)	0.64 (0.42)
		Median	1.00	0.00	0.00	1.14	0.00	0.36	0.63
		Q1–Q3	0.25–1.75	0.00–0.67	0.00–0.67	0.43–1.86	0.00–0.00	0.18–0.73	0.38–0.78
		Min–Max	0.0–3.0	0.0–3.0	0.0–1.3	0.0–3.1	0.0–1.5	0.0–1.9	0.0–1.8
	Day 15	N with data	36	35	36	36	36	36	36
		Mean (SD)	0.64 (0.72)	0.30 (0.54)	0.19 (0.32)	0.79 (0.85)	0.14 (0.45)	0.37 (0.61)	0.44 (0.52)
		Median	0.50	0.00	0.00	0.50	0.00	0.18	0.25
		Q1–Q3	0.00–1.00	0.00–0.33	0.00–0.33	0.00–1.43	0.00–0.00	0.00–0.36	0.08–0.67
		Min–Max	0.0–2.3	0.0–2.0	0.0–1.0	0.0–3.1	0.0–2.3	0.0–3.1	0.0–2.3
	Maximum score	N with data	37	37	37	37	37	37	NA
		Mean (SD)	1.66 (0.99)	1.37 (1.03)	0.81 (0.89)	1.82 (0.83)	0.26 (0.48)	1.20 (0.85)	NA
		Median	1.75	1.33	0.67	1.71	0.00	1.00	NA
		Q1–Q3	1.00–2.25	0.67–2.00	0.00–1.00	1.14–2.43	0.00–0.25	0.55–1.82	NA
		Min–Max	0.0–3.8	0.0–4.0	0.0–3.0	0.4–3.7	0.0–2.3	0.1–3.2	NA
65–69 YOA	Day 1	N with data	36	36	36	36	36	36	36
		Mean (SD)	1.80 (1.02)	1.25 (1.03)	0.77 (1.00)	1.82 (0.82)	0.11 (0.20)	0.92 (0.69)	1.14 (0.55)
		Median	1.88	1.00	0.33	1.57	0.00	0.82	0.98
		Q1–Q3	1.00–2.38	0.33–2.00	0.00–1.33	1.14–2.36	0.00–0.25	0.32–1.32	0.66–1.47
		Min–Max	0.0–4.0	0.0–3.5	0.0–4.0	0.6–4.0	0.0–0.8	0.0–2.8	0.5–2.8
	Day 8	N with data	35	35	35	35	35	35	35
		Mean (SD)	1.23 (1.01)	0.57 (0.81)	0.40 (0.68)	1.25 (0.98)	0.11 (0.25)	0.58 (0.58)	0.73 (0.59)
		Median	1.25	0.33	0.00	1.00	0.00	0.45	0.72
		Q1–Q3	0.25–2.00	0.00–0.67	0.00–0.67	0.43–2.00	0.00–0.00	0.09–0.82	0.28–1.09
		Min–Max	0.0–3.5	0.0–3.0	0.0–3.0	0.0–3.4	0.0–1.0	0.0–2.5	0.0–2.3
	Day 15	N with data	34	33	34	34	34	34	34
		Mean (SD)	0.55 (0.77)	0.30 (0.77)	0.33 (0.85)	0.59 (0.81)	0.07 (0.19)	0.34 (0.62)	0.39 (0.60)
		Median	0.25	0.00	0.00	0.29	0.00	0.05	0.09
		Q1–Q3	0.00–0.75	0.00–0.00	0.00–0.33	0.00–0.86	0.00–0.00	0.00–0.45	0.03–0.56
		Min–Max	0.0–3.8	0.0–4.0	0.0–4.0	0.0–3.0	0.0–0.8	0.0–2.6	0.0–2.5
	Maximum score	N with data	36	36	36	36	36	36	NA
		Mean (SD)	1.99 (0.95)	1.40 (1.06)	1.00 (1.10)	1.92 (0.82)	0.22 (0.29)	0.96 (0.69)	NA
		Median	2.00	1.33	0.67	1.71	0.00	0.82	NA
		Q1–Q3	1.50–2.50	0.67–2.00	0.00–1.50	1.31–2.43	0.00–0.38	0.45–1.36	NA
		Min–Max	0.0–4.0	0.0–4.0	0.0–4.0	0.6–4.0	0.0–1.0	0.0–2.8	NA
70–79 YOA	Day 1	N with data	45	45	45	45	45	45	45
		Mean (SD)	1.48 (0.97)	1.08 (1.06)	0.53 (0.94)	1.72 (0.67)	0.11 (0.24)	0.87 (0.73)	1.02 (0.52)
		Median	1.33	1.00	0.00	1.67	0.00	0.73	0.84
		Q1–Q3	0.75–2.00	0.00–1.33	0.00–0.67	1.14–2.29	0.00–0.00	0.27–1.27	0.69–1.41
		Min–Max	0.0–3.5	0.0–4.0	0.0–3.0	0.6–3.0	0.0–1.0	0.0–2.7	0.1–2.2
	Day 8	N with data	44	44	44	44	44	44	44
		Mean (SD)	0.56 (0.63)	0.21 (0.37)	0.23 (0.52)	0.97 (0.65)	0.12 (0.34)	0.35 (0.38)	0.46 (0.36)
		Median	0.50	0.00	0.00	0.86	0.00	0.27	0.39
		Q1–Q3	0.00–1.00	0.00–0.33	0.00–0.33	0.57–1.50	0.00–0.00	0.00–0.55	0.22–0.52
		Min–Max	0.0–2.3	0.0–1.3	0.0–2.3	0.0–2.4	0.0–2.0	0.0–1.8	0.0–1.8
	Day 15	N with data	45	45	45	45	45	45	45
		Mean (SD)	0.31 (0.52)	0.13 (0.36)	0.13 (0.28)	0.50 (0.57)	0.08 (0.23)	0.16 (0.23)	0.24 (0.27)
		Median	0.00	0.00	0.00	0.29	0.00	0.00	0.19
		Q1–Q3	0.00–0.50	0.00–0.00	0.00–0.00	0.00–0.86	0.00–0.00	0.00–0.27	0.00–0.31
		Min–Max	0.0–2.0	0.0–2.0	0.0–1.3	0.0–2.0	0.0–1.3	0.0–0.9	0.0–1.2
	Maximum score	N with data	45	45	45	45	45	45	NA
		Mean (SD)	1.60 (0.95)	1.11 (1.05)	0.64 (0.96)	1.78 (0.62)	0.23 (0.41)	0.91 (0.70)	NA
		Median	1.50	1.00	0.00	1.71	0.00	0.73	NA
		Q1–Q3	0.75–2.00	0.33–1.33	0.00–0.67	1.29–2.29	0.00–0.25	0.36–1.27	NA
		Min–Max	0.0–3.5	0.0–4.0	0.0–3.0	0.6–3.0	0.0–2.0	0.0–2.7	NA
≥80 YOA	Day 1	N with data	17	17	16	17	17	17	17
		Mean (SD)	1.50 (1.04)	1.08 (1.17)	0.56 (0.99)	1.53 (0.79)	0.16 (0.38)	0.87 (0.63)	1.00 (0.53)
		Median	1.50	1.00	0.00	1.29	0.00	1.00	1.13
		Q1–Q3	0.75–2.00	0.00–1.33	0.00–0.83	1.00–2.14	0.00–0.00	0.18–1.18	0.69–1.26
		Min–Max	0.0–3.5	0.0–4.0	0.0–3.3	0.0–3.0	0.0–1.3	0.0–2.1	0.1–2.0
	Day 8	N with data	18	18	18	18	18	18	18
		Mean (SD)	0.82 (0.76)	0.28 (0.56)	0.35 (0.59)	0.95 (0.60)	0.07 (0.19)	0.48 (0.45)	0.54 (0.38)
		Median	0.75	0.00	0.00	0.86	0.00	0.36	0.40
		Q1–Q3	0.00–1.50	0.00–0.33	0.00–0.33	0.57–1.43	0.00–0.00	0.09–0.82	0.23–0.84
		Min–Max	0.0–2.3	0.0–2.0	0.0–2.0	0.0–1.9	0.0–0.8	0.0–1.4	0.1–1.3
	Day 15	N with data	18	18	18	18	18	18	18
		Mean (SD)	0.41 (0.56)	0.11 (0.20)	0.22 (0.50)	0.39 (0.58)	0.03 (0.12)	0.17 (0.30)	0.23 (0.33)
		Median	0.25	0.00	0.00	0.14	0.00	0.05	0.11
		Q1–Q3	0.00–0.75	0.00–0.33	0.00–0.00	0.00–0.43	0.00–0.00	0.00–0.18	0.00–0.25
		Min–Max	0.0–2.0	0.0–0.7	0.0–1.7	0.0–1.7	0.0–0.5	0.0–1.2	0.0–1.2
	Maximum score	N with data	18	18	18	18	18	18	NA
		Mean (SD)	1.63 (0.93)	1.07 (1.12)	0.63 (0.92)	1.67 (0.67)	0.24 (0.40)	0.95 (0.56)	NA
		Median	1.63	0.83	0.33	1.71	0.00	1.05	NA
		Q1–Q3	1.00–2.00	0.00–1.33	0.00–1.00	1.14–2.14	0.00–0.50	0.55–1.18	NA
		Min–Max	0.3–3.5	0.0–4.0	0.0–3.3	0.7–3.0	0.0–1.3	0.0–2.1	NA

Abbreviations: cRSV-ARI, confirmed respiratory syncytial virus-associated acute respiratory infection; FLU-PRO, InFLUenza Patient-Reported Outcome; Max, maximum; Min, minimum; N, number of participants; NA, not available; Q1, 25% percentile; Q3, 75% percentile; SD, standard deviation; YOA, years of age.

**Figure 4.**
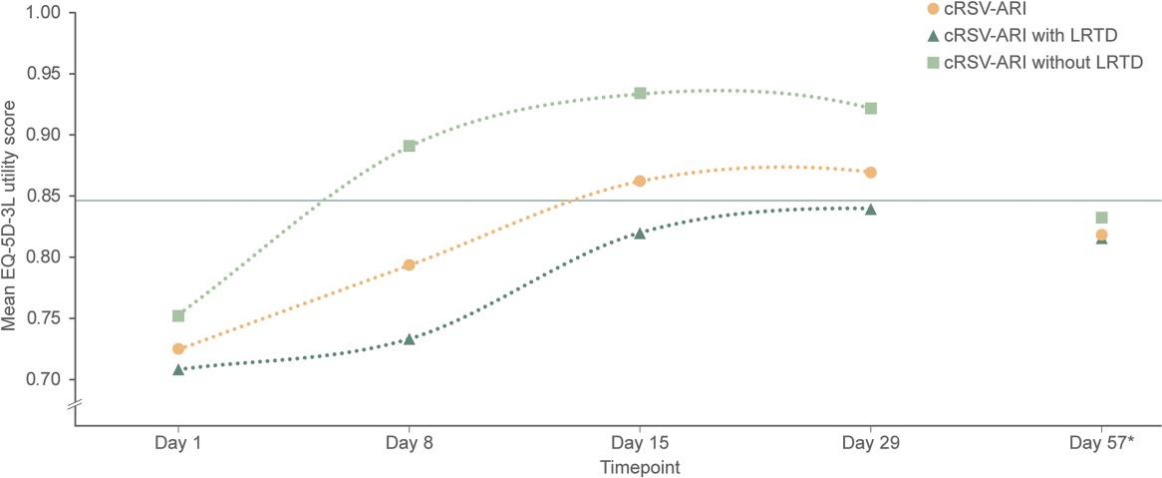
Mean EQ-5D-3L utility score at Days 1, 8, 15, 29, and 57 by LRTD status for participants with a medically-attended cRSV-ARI (analysis set). Abbreviations: cRSV-ARI, confirmed respiratory syncytial virus-associated acute respiratory infection; EQ-5D-3L, EuroQoL-5 Dimension; LRTD; lower respiratory tract disease; UK TTO, United Kingdom Time-Trade-Off. *Only for participants with symptoms persisting after Day 29. The mean EQ-5D utility score was scored using UK TTO [21]. EQ-5D (UK TTO) scores range from −0.594 to 1, with a higher value indicating a higher level of functioning or of quality of life. The horizontal line represents the weighted baseline value (0.8466) calculated based on the baselines reported in the literature [21] and the number of participants in each age category in the present study.

The mean cRSV-ARI EQ-5D-3L utility scores in participants with a medically-attended cRSV-ARI were 0.81 (SD: 0.22) on Day 1, 0.85 (0.21) on Day 8, 0.89 (0.22) on Day 15, 0.90 (0.21) on Day 29, and 0.84 (0.31) on Day 57. On Day 1, the cRSV-ARI utility scores in participants with a medically-attended cRSV-ARI in the different age groups were: 0.80 (SD: 0.24) in the 60–64 YOA group, 0.86 (0.13) in the 65–69 YOA group, 0.80 (0.24) in the 70–79 YOA group, and 0.72 (0.27) in the ≥80 YOA group (Table 4). A trend for an increased effect on HRQoL was observed when comparing participants with cRSV-ARI LRTD and those without cRSV-ARI LRTD (Figure 4).

**Table 4.** EQ-5D-3L Utility Score at Days 1, 8, 15, 29, and 57^a^ for Participants with a Medically-Attended cRSV-ARI by Additional Age Group and Overall (Analysis Set for Medically-Attended cRSV-ARI Cases)

_Age Category		Age category				
		60–64 YOA*N* = 37	65–69 YOA*N* = 36	70–79 YOA*N* = 45	≥80 YOA*N* = 18	Total*N* = 136
Day 1	N with data	37	35	45	17	134
	Mean (SD)	0.7981 (0.2407)	0.8636 (0.1299)	0.8013 (0.2362)	0.7228 (0.2697)	0.8067 (0.2214)
	Median	0.8680	0.8870	0.8600	0.7880	0.8680
	Q1–Q3	0.7810 to 1.0000	0.7960 to 1.0000	0.7810 to 0.9250	0.6410 to 0.8870	0.7810 to 0.9250
	Min–Max	−0.1810 to 1.0000	0.3600 to 1.0000	−0.0800 to 1.0000	0.0300 to 1.0000	−0.1810 to 1.0000
Day 8	N with data	35	35	45	18	133
	Mean (SD)	0.7745 (0.3251)	0.8893 (0.1391)	0.8884 (0.1119)	0.8242 (0.2306)	0.8500 (0.2139)
	Median	0.8870	0.8940	0.8940	0.8870	0.8870
	Q1–Q3	0.7700 to 1.0000	0.8160 to 1.0000	0.8500 to 1.0000	0.7200 to 1.0000	0.7960 to 1.0000
	Min–Max	−0.1810 to 1.0000	0.3460 to 1.0000	0.5140 to 1.0000	0.0300 to 1.0000	−0.1810 to 1.0000
Day 15	N with data	36	34	44	18	132
	Mean (SD)	0.8394 (0.3550)	0.9290 (0.1062)	0.9249 (0.1213)	0.8511 (0.2409)	0.8926 (0.2249)
	Median	1.0000	1.0000	1.0000	0.8870	1.0000
	Q1–Q3	0.8775 to 1.0000	0.8700 to 1.0000	0.8870 to 1.0000	0.7960 to 1.0000	0.8700 to 1.0000
	Min–Max	−0.3490 to 1.0000	0.6560 to 1.0000	0.5140 to 1.0000	−0.0160 to 1.0000	−0.3490 to 1.0000
Day 29	N with data	34	35	42	15	126
	Mean (SD)	0.8494 (0.2989)	0.9334 (0.0966)	0.9166 (0.1763)	0.8666 (0.2773)	0.8972 (0.2148)
	Median	1.0000	1.0000	1.0000	1.0000	1.0000
	Q1–Q3	0.8870 to 1.0000	0.8870 to 1.0000	0.8870 to 1.0000	0.8000 to 1.0000	0.8870 to 1.0000
	Min–Max	−0.1840 to 1.0000	0.7100 to 1.0000	0.1410 to 1.0000	−0.0740 to 1.0000	−0.1840 to 1.0000
Day 57^a^	N with data	8	6	8	2	24
	Mean (SD)	0.7644 (0.4419)	0.9527 (0.1159)	0.9466 (0.0785)	0.4040 (0.4469)	0.8422 (0.3142)
	Median	1.0000	1.0000	1.0000	0.4040	1.0000
	Q1–Q3	0.5945 to 1.0000	1.0000 to 1.0000	0.8870 to 1.0000	0.0880 to 0.7200	0.8430 to 1.0000
	Min–Max	−0.0740 to 1.0000	0.7160 to 1.0000	0.7990 to 1.0000	0.0880 to 0.7200	−0.0740 to 1.0000

Abbreviations: cRSV-ARI, confirmed respiratory syncytial virus-associated acute respiratory infection; EQ-5D-3L, EuroQoL-5 Dimension; Max, maximum; Min, minimum; N, number of participants; Q1, 25% percentile; Q3, 75% percentile; SD, standard deviation; YOA, years of age.

^a^Only for participants with symptoms persisting after Day 29.

### HCRU and Working Days Lost by Participants with Medically-attended cRSV-ARI and Their Caregivers

Of the 136 participants with a medically-attended cRSV-ARI, 126 (92.6%) visited their GP at least once during an ARI episode, with a mean number of GP visits of 1.33. The mean number of GP visits for participants with a medically-attended cRSV-ARI in the different age groups was: 1.24 in the 60–64 YOA group, 1.36 in the 65–69 YOA group, 1.18 in the 70–79 YOA group, and 1.83 in the ≥80 YOA group. Twenty-three (16.9%) participants with a medically-attended cRSV-ARI visited the ER and four (2.9%) visited a pulmonologist. A total of 120 (88.2%) participants with a medically-attended cRSV-ARI took any medication, with 88 (64.7%) taking any antibiotic and 55 (40.4%) taking any antipyretic. The mean duration of treatment was 16.3 (SD: 11.9) days. One participant (0.7%) was hospitalized for 14 days, and none were admitted to the ICU. Five out of the 18 participants reporting to be in active employment (27.8%) stayed home from work, with a mean productivity loss of 7.0 (SD: 3.6) days. Caregivers of participants with a medically-attended cRSV-ARI were not absent for work.

## DISCUSSION

Recently, a phase 3 trial in adults ≥60 YOA showed that a licensed RSV vaccine reduced RSV symptom severity in breakthrough infections, with a trend toward a reduced impact on physical functioning and health utility [22]. The methodology we used for the present study was similar and included participants who were not vaccinated against RSV. The present study was designed as an outpatient study but not specifically designed to exclude more severe RSV cases. However, our study is not representative for more severe cases. These were likely missed if for example, patients did not visit their GP or outpatient clinic prior to an ER visit or hospitalization.

In this study, we demonstrated a substantial symptom duration (median of 2.0–19.0 days for any symptom). Data found in the literature report a maximum duration of illness of 7–10 days depending on the season [23], which is shorter compared to the present study; however, other studies reported a median symptom duration of 17 days [17] and a mean symptom duration of 19 days [24], comparable to the symptom duration reported in the present study.

Moderate to severe symptoms were observed in participants with a medically-attended cRSV-ARI during the first seven days of the ARI episode. The chest/respiratory and the nose domains were the most affected by medically-attended cRSV-ARIs, while age did not have an influence on the scores. Studies using the FLU-PRO questionnaire have also been conducted in individuals infected with other respiratory viruses. In a human challenge model among adults 18–40 YOA in the United States, participants were asked to complete the FLU-PRO questionnaire after intranasal challenge with a wild-type influenza strain. Peak FLU-PRO scores occurred on Day 3 postinoculation and were highest for the nose, body/systemic, and throat domains [25]. In a longitudinal study in ten military treatment facilities in the United States, participants with SARS-CoV-2 infection completed ≥1 FLU-PRO Plus survey within two weeks after symptom onset. Peak FLU-PRO scores in participants were reported for the nose, body/systemic, and respiratory domains [26]. Although the populations and the study design differ, these results indicate that symptomology is different for cRSV-ARI, compared to other common respiratory diseases.

In the present study, a negative effect on HRQoL was observed in the cRSV-ARI utility score at Day 1 when compared to the scores at Day 29 for participants with a medically-attended cRSV-ARI. The effect on HRQoL was greater for participants with LRTD compared to those without LRTD. In a recent study conducted among European community-dwelling older adults, the utility value decreased from 0.90 preseason to 0.80 one week after symptom onset [9]. In the present study, the mean cRSV-ARI utility score was 0.81 on Day 1. Although the baseline value was not collected during the present study and the study designs differed, the utility scores are comparable [9]. When comparing the scores with the baseline reported in the literature (weighted baseline calculated based on the baselines reported in the literature [21] and the number of participants in each age category in the present study: 0.85), our results show that medically-attended cRSV-ARIs have an effect on HRQoL as the scores at Day 1 are lower than the baseline reported in the literature [21]. Moreover, a recent cross-sectional study in US adults ≥60 YOA found that they were willing to trade 4.9–9.1 days of their life to avoid upper respiratory tract disease, LRTD, or severe LRTD at an older age [27]. These results indicate that RSV affects the QoL of older adults.

Most participants with a medically-attended cRSV-ARI visited their GP, and 27.8% of participants in active employment were absent for work. As not all older adults are employed, nonmarket productivity losses should be considered. A recent study estimated that RSV in older adults caused approximately US$4.7 billion in productivity losses each year [28], indicating that RSV has an important impact in this population. As we showed that medically-attended cRSV-ARI episodes affect European adults ≥60 YOA who were not vaccinated against RSV, this study may help inform decisions about interventions to prevent RSV in older adults.

In addition to the limitations mentioned in Terns Riera et al [15], the study included a limited number of medically-attended ARI cases as not all individuals with an ARI seek medical advice, especially if the symptoms are mild. Moreover, ARI cases may have been missed if individuals experienced a single symptom or if the first symptom occurred ≥7 days (for RSV season 1) or ≥10 days (after RSV season 1) before Day 1. This may have led to an under- or overestimation of medically-attended RSV-ARI cases. There may also be a selection bias as individuals who consent to study participation may be different from those who do not, and individuals with a cognitive disability were excluded. Lastly, as the EQ-5D baseline (ie, the health status before the cRSV-ARI episode) could not be collected due to recruitment after onset of ARI symptoms, the baseline was based on results from the literature which included the five largest European economies (ie, France, Germany, Italy, Spain, and the United Kingdom) [21]. As no baseline utility estimates were collected, quality-adjusted life years and quality-adjusted life days lost could not be estimated. This also complicates determining whether patients return to their baseline health status, but the results of the present study can inform on the patient's pace of recovery.

The study population may not be entirely representative for the overall population studied due to the study site selection, the mandatory swabs, the inclusion of private healthcare providers, and the exclusion of people with a cognitive disability. Moreover, most participants in the present study were White.

The study also has several strengths in addition to the ones mentioned in Terns Riera et al [15]. The data were collected prospectively and with a specific data collection method, with the aim of answering specific research questions, leading to a more complete dataset. Another strength of this study was the high compliance with questionnaire completion, leading to a more complete dataset and less likely introduction of bias.

In conclusion, medically-attended cRSV-ARIs substantially affect the symptomology, especially the chest/respiratory and the nose domains, and the HRQoL in European adults ≥60 YOA who were not vaccinated against RSV.
